# Letter to the Editor: ‘Using data and artificial intelligence to improve care pathways of older people experiencing falls and frailty: Opportunities, challenges and practical considerations for clinicians’

**DOI:** 10.1016/j.clinme.2026.100586

**Published:** 2026-04-15

**Authors:** Preeti Lamba

**Affiliations:** Dr. D. Y. Patil Vidyapeeth (Deemed to be University), Pimpri, Pune, Maharashtra, India

To the Editor,

I read with interest the recent article by Chan, which provides a timely overview of artificial intelligence (AI) and machine learning (ML) in falls and frailty care among older adults. The article appropriately highlights translational gaps and ethical concerns, such as digital ageism. However, several methodological aspects merit further clarification to strengthen clinical applicability.^1^

First, although AI-based prediction models are well summarised, standardised evaluation metrics such as calibration and clinical utility are not adequately addressed. Evidence suggests that while many ML models demonstrate acceptable discrimination, they often exhibit poor calibration and high risk of bias, limiting their real-world usefulness.^2^

Second, generalisability across healthcare settings remains a critical concern. Models trained on localised datasets frequently underperform in external populations due to variations in data quality, coding practices and sociodemographic characteristics. Addressing dataset heterogeneity and missing data is essential for improving robustness and external validity.^3^

Third, implementation challenges such as clinician trust, alert fatigue and workflow integration are underexplored. Real-world studies indicate that predictive performance alone does not translate into improved outcomes unless models are effectively embedded into clinical decision pathways.^4^

Fourth, while digital ageism is acknowledged, the role of explainable AI requires greater emphasis. Transparency and interpretability are crucial for clinician acceptance and safe decision-making, particularly in frail older populations.^5^

Finally, despite noting limited randomised evidence, stronger emphasis on prospective validation is warranted. Recent trials of AI-based clinical decision support systems demonstrate mixed outcomes, underscoring the gap between predictive accuracy and clinical effectiveness.^6^

In summary, this article provides a valuable overview of AI in geriatric care. Greater focus on standardised evaluation, generalisability, explainability and implementation science would enhance its translational impact. Robust multicentre prospective studies are essential for safe and equitable integration of AI into clinical practice.

## CRediT authorship contribution statement

**Preeti Lamba:** Conceptualization, Writing – original draft, Writing – review & editing.

## Funding

No funding was received for the preparation of this letter to the Editor. The author did not receive any financial support from public, commercial or not-for-profit funding agencies for the research, authorship, or publication of this work.

## Declaration of competing interest

The authors declare that they have no known competing financial interests or personal relationships that could have appeared to influence the work reported in this paper.
